# Health status and genetic compositions of green turtles (*Chelonia mydas*) foraging in Brunei Bay

**DOI:** 10.1371/journal.pone.0293979

**Published:** 2023-11-09

**Authors:** Juanita Joseph, Hideaki Nishizawa, Siti Nabilla Jalimin, Rafidah Othman, Saifullah Arifin Jaaman, James Bali, Zhang Xuelei

**Affiliations:** 1 Borneo Marine Research Institute, Universiti Malaysia Sabah, Kota Kinabalu, Sabah, Malaysia; 2 Institute of Oceanography and Environment, Universiti Malaysia Terengganu, Kuala Terengganu, Malaysia; 3 Graduate School of Informatics, Kyoto University, Kyoto, Japan; 4 Protected Areas & Biodiversity Conservation Division, Sarawak Forestry Corporation Sdn. Bhd., Kota Sentosa, Kuching, Sarawak. Malaysia; 5 MNR Key Laboratory of Marine Eco-Environmental Science and Technology, First Institute of Oceanography, Ministry of Natural Resources (MNR), Qingdao, China; MARE – Marine and Environmental Sciences Centre, PORTUGAL

## Abstract

Long-term monitoring of sea turtle aggregations is critical for understanding the impacts of environmental changes on their population health and habitat suitability. Brunei Bay is a significant foraging ground for green turtles in the South China Sea. We analyzed the body size, hematology and body condition of green turtles for their health status in their foraging ground in Brunei Bay over a period of nine years (2011–2019). Additionally, we used mitochondrial DNA (mtDNA) to evaluate changes in the size and genetic composition of green turtle aggregations. Our findings revealed that the size composition of the green turtle population varied seasonally, but there were no significant temporal changes in genetic and size compositions. Hematology parameters and Fulton’s body condition index were consistent with those reported for apparently healthy green turtles. Furthermore, we found that blood reference intervals indicated the turtles were healthy. These results provide valuable baseline data for future comparisons with other foraging aggregations and for long-term monitoring of green turtles in Brunei Bay.

## Introduction

Foraging grounds constitute a crucial habitat for the growth of sea turtles [1]. Among the sea turtle species, the green turtle (*Chelonia mydas*) primarily relies on coastal areas for foraging and primarily consumes seagrass or algae [2]. However, coastal regions are highly susceptible to pollution and human activities, leading to several threats to green turtles, such as deteriorating water quality [3], exposure to biotoxins produced by harmful algal blooms [4], infectious diseases such as fibropapilomatosis [5, 6], human-induced disturbances such as marine debris [7], and boating accidents [8]. Therefore, it is imperative to comprehend the health status and body condition of green turtles inhabiting foraging grounds to conserve and evaluate their foraging habitats [9].

The assessment of sea turtle health using tools such as the body condition index, which is calculated through morphometric measurements [10, 11], and hematology parameters obtained from blood samples [12, 13], are valuable indicators. However, regional, and ontogenetic differences necessitate the establishment of baseline and reference intervals to monitor sea turtle populations effectively [14–17]. Additionally, the prevalence of fibropapilloma [18] or epibiotic load [19, 20], and their relationship with hematology parameters, provide insight into the overall health of sea turtle populations. Given the green turtle’s long lifespan and delayed maturation, establishing a baseline health status for this species, and monitoring it over time is critical.

Brunei Bay, situated in the South China Sea, is an important foraging ground for green turtles [21], accommodating a considerable population of juvenile and adult turtles that originate from Southeast Asian rookeries, particularly the Sulu Sea population [21, 22]. The bay’s rich marine ecosystem supports diverse wildlife, including the dugong, *Dugong dugon* [23] and Irrawaddy dolphins, *Orcaella brevirostris* [24], which forage in the region. Despite the presence of contaminants at relatively low levels [25, 26], the activities of humans and industries, such as microplastics, remain a concern [27]. Moreover, the Brunei Bay region, particularly within Sarawak, Malaysia, faces the potential development of petrochemical and refinery plants, further increasing the risks to the area’s ecosystem [28].

The present study aims to establish reference intervals for the body condition and health status of the green turtle foraging aggregation in Brunei Bay. Furthermore, this study investigates seasonal and temporal changes in size and genetic compositions relative to previous studies [21, 22]. While Ihwan et al. [29] previously reported the presence of the barnacle *Chelonibia testudinaria* on green turtles in Brunei Bay, no information on the body condition and health status of the turtles has been reported. In this study, we report the hematology parameters of the Brunei Bay green turtle aggregation. Size and genetic composition data were obtained from a survey conducted during 2011–2016 [21, 22], and we extend this data set by including survey data from 2018–2019 to assess the stability of the Brunei Bay aggregation composition over time.

## Materials and methods

This study strictly adhered to the recommendations provided by the Sarawak Forestry Department under Permit No. (139) JHS/NCCD/600-7/2/107/Jld2. No surgeries, anesthesia, or animal sacrifice were involved in this study. Blood withdrawals were conducted according to standard procedures and were carried out only by trained researchers.

### Study site

**Fig 1:**
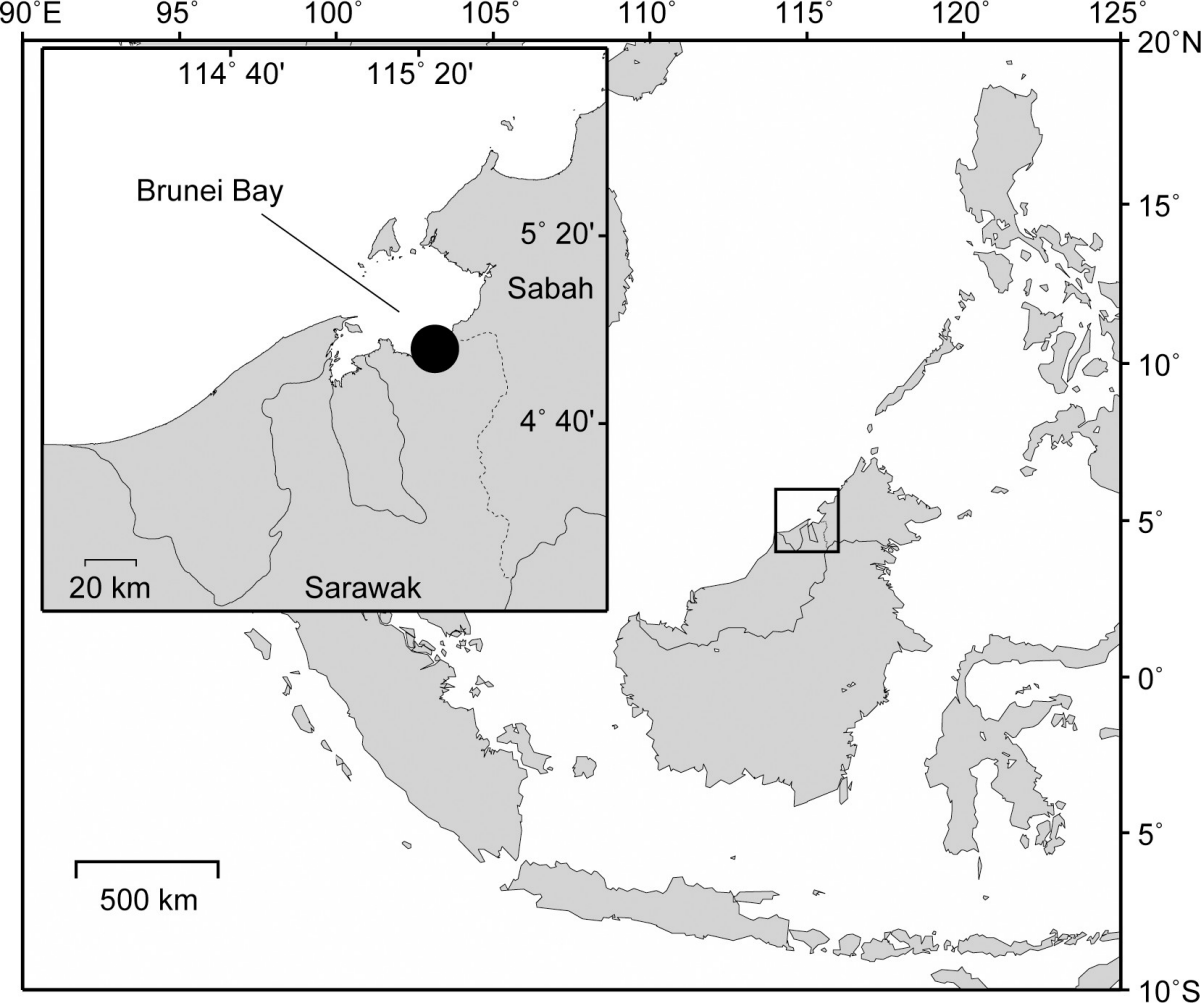
Map of Brunei Bay, Lawas, Sarawak, Malaysia. The map was created using the Maptool program of SEATURTLE.ORG.

The study was conducted in Lawas, Sarawak (4°45’ N–5°02’ N, 114°58’ E–115°10’ E), located on the north-western coast of Borneo Island (Fig 1). In addition to utilizing data collected during 2011 to 2016 [21, 22], we collected new samples during 2018 and 2019. Sampling trips lasted for 2–3 days and coincided with the spring tide. Sampling occurred four times across 2018 and 2019: in January, April, August 2018, and in January 2019. The sea turtles were captured using a traditional barrier net known as Kabat, which is the same sea turtle catching technique used by Joseph et al. [21]. The Kabat was set during the highest tide of each month and captured sea turtles were checked for the following day during the lowest tide.

### Fieldwork and sample collection

A total of 117 green turtles were collected during six sampling trips conducted in 2011, 2013, 2014, 2016, 2018, and 2019. Each turtle was measured for curved carapace length (CCL) and curved carapace width (CCW) using a flexible measuring tape (±0.1 cm). The size classification of green turtles followed the criteria proposed by Sterling et al. [30], where turtles with a CCL of <65.0 cm were considered juveniles, those with a CCL of 65.0–85.0 cm were considered sub-adults, and those with a CCL of >85.0 cm were considered adults. Body weight was measured using a spring scale (±0.1 kg). The sex of sea turtles was determined externally, which could only be done for mature turtles. Males were identified by the presence of a tail that protrudes above the carapace [31].

Blood samples (3 mL) were collected by venipuncture from the cervical venous sinus after manual fixation with sterilized 21-gauge needles and 5 mL syringes. Blood samples collected in 2018 and 2019 (*N* = 60) were transferred to BD Vacutainer© K2 tubes containing ethylenediaminetetraacetic acid (EDTA) for subsequent complete blood count analysis. All samples were stored at -4°C until further analysis. Blood samples for genetic analysis were stored in a lysis buffer following standard protocols [21].

### Health and hematology assessments

The Fulton’s body condition index (BCI) is used to determine the nutritional status and energy reserves and was calculated using the formula BCI = [weight (kg) / SCL^3^ (m)] × 10,000 [32], where SCL indicates straight carapace length. We converted CCL to SCL by the equation of Teas [33].

External tumours were visually inspected, and tumour diameter and number were recorded for each turtle. Tumour scores were assigned based on Work and Balazs [34], with scores of 0, 1, 2, and 3 representing no symptoms, mild, moderate, and severe symptoms, respectively. Additionally, in 2018 and 2019, epibionts were collected from the turtles following the methods described in Pinou et al. [35]. The number of barnacles on each turtle was recorded.

The blood samples were sent to a commercial lab (Quantum Diagnostics Commercial Laboratory, Kota Kinabalu, Sabah) for hematology analysis. Each sample was analyzed in triplicate analysis. A total of 13 parameters were measured including red blood cell (RBC), hemoglobin (HB), hematocrit (HCT), mean cell volume (MCV), mean cell hemoglobin (MCH), mean cell hemoglobin concentration (MCHC), white blood cell (WBC), heterophils (HET), lymphocytes (LYM), monocytes (MO), eosinophils (EO), basophils (BA), and thrombocytes (TH). In addition, we calculated ratio of HET to LYM (H: L ratio) that is closely related to stress hormones [36].

### Genetic analysis

Genetic analysis was conducted to identify the haplotype composition of the newly collected samples at the Fish Disease Laboratory of the Borneo Marine Research Institute, Universiti Malaysia Sabah. DNA extraction was carried out using the Vivantis GF-1 Tissue Blood Combi DNA Extraction Kit, with minor modifications to suit the sea turtle blood sample. The mitochondrial control region (~770bp) was amplified using primers LCM15382 and H950g, following the protocol of Joseph et al. [21]. Amplification by polymerase chain reaction (PCR) was performed in a 50 μl reaction volume containing 25 to 50 ng of sea turtle genomic DNA, 1U/50 μl Taq polymerase, 10 mM TBE buffer, 2.5 mM magnesium chloride MgCl_2_, 0.125 mM deoxynucleotide triphosphates (dNTPs) and 0.2 μM of each primer.

The PCR cycling parameters were as follows: an initial denaturation at 94°C for 3 mins, followed by 30 cycles of denaturation at 94°C for 30 sec, annealing at 55°C for 30 sec, and extension at 72°C for 60 sec, followed by a final elongation step at 72°C for 3 mins. PCR was carried out using the Applied Biosystems thermal cycler (model AB venti) and standard precautions were taken to prevent contamination during PCR preparation. The PCR products were sent to Next Gene for PCR purification and sequencing of both strands. The resulting sequences were analyzed using Chromas ver 2.6.6 and aligned using MEGA software ver 11 [37].

Haplotype composition was determined by searching the database for known green sea turtle haplotypes from the Pacific and Indian Oceans, which was done by reviewing mtDNA sequences published on the Southwest Fisheries Science Centre, NOAA Fisheries Service website and the GenBank database (National Centre for Biotechnology Information, USA: NCBI website http://www.ncbi.nlm.nih.gov) for sequence comparisons of the control region.

### Data analysis

Seasonal difference in size composition was tested by comparing CCL data among January (*N* = 17), April (*N* = 17), and August (*N* = 22) in 2018 and January 2019 (*N* = 5). In addition, we tested the difference in CCL of green turtles captured in December-May among three categories of years: 2011 (*N* = 28), 2013–2014 (*N* = 19), and 2018–2019 (*N* = 39). In this analysis, we excluded data from August 2018 because the CCL was significantly lower than in January or April (see Results). Differences were tested by pairwise Wilcoxon rank sum tests using rstatix package in R ver 4.1.3 [38].

Haplotypes frequencies of Brunei Bay aggregation were compared among January (*N* = 17), April (*N* = 17), and August (*N* = 19) in 2018 and January 2019 (*N* = 5) by exact tests (50,000 iterations in a Markov chain with a 10,000-iteration dememorization) implemented in Arlequin ver 3.5 [39]. Temporal changes were also tested by comparing 2011 (*N* = 23), 2013–2014 (*N* = 19), and 2018–2019 (*N* = 61), part of which was previously reported in Joseph et al. [21]. Then, overall haplotypes frequencies were also compared between immature (juvenile and subadult turtles; CCL < 85.0 cm) and mature turtles (CCL > 85.0 cm). The natal origin of immature and mature turtles was estimated by Bayesian mixed stock analysis (MSA) using BAYES software [40]. The analysis followed the settings of Nishizawa et al. [22], but Xisha (Paracel) Islands [41] were added to candidate sources that contained 32 management units in total from the eastern Indian Ocean and southern Pacific that were grouped into 5 regions (southern Pacific, Micronesia, South China Sea, Sulu and Celebes Sea, and eastern Indian Ocean). Both uniform prior distribution and prior distribution weighted by the number of nests in each rookery were considered.

Correlations between blood parameters and CCL were tested by Kendall rank correlation coefficients. Correlations between blood parameters and the number of barnacles were also tested. The relationship between the number of barnacles and CCL was tested by linear modeling after the log transformation of the number of barnacles. Values lower than the limit of detection (LOD) were submitted by LOD /√2 [42]. Values of basophils (BA) were lower than LOD in most turtles; thus, we removed BA from the analyses. Differences in BCI between maturity stages were tested by the chi-square test.

The significant difference is defined at p-value < 0.05. In pairwise comparisons and correlations of blood parameters, the significance threshold was subsequently adjusted by sequential Bonferroni correction.

## Results

### Size class distribution

**Fig 2:**
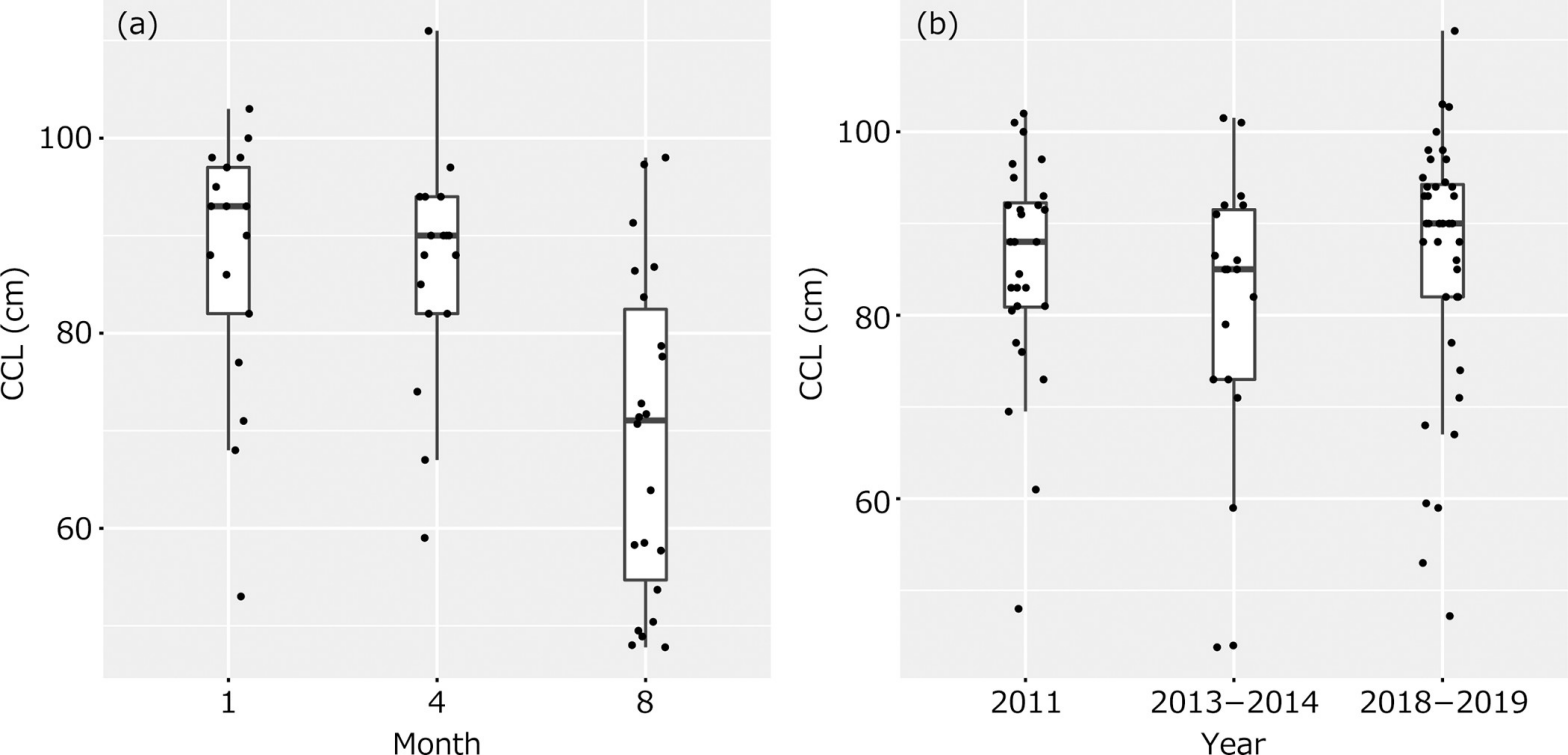
Boxplots with individual data points of CCL of captured green turtles in (a) January, April, and August in 2018 and January 2019, and (b) different years.

In total, we captured 117 green turtles between 2011 and 2019, except for one hawksbill turtle that was found in 2017 [43]. The mean CCL and CCW of the 116 green turtles measured were 82.4 ± 15.8 cm (range = 43.8 to 111.0 cm) and 74.7 ± 14.5 cm (range = 40.5 to 96.0 cm), respectively, with one individual not measured. Notably, the CCL of green turtles captured in August 2018 was significantly lower than that in January (Z = 3.145, p = 0.002) or April (Z = 3.146, p = 0.002) (Fig 2A). However, there was no evidence of a temporal trend in CCL over the years (Z < 1.061, p > 0.289) (Fig 2B). The sex of the green turtles was 35.9% immature turtle, 59% adult female turtle, and 5.1% adult male turtle.

### Health assessments

**Fig 3:**
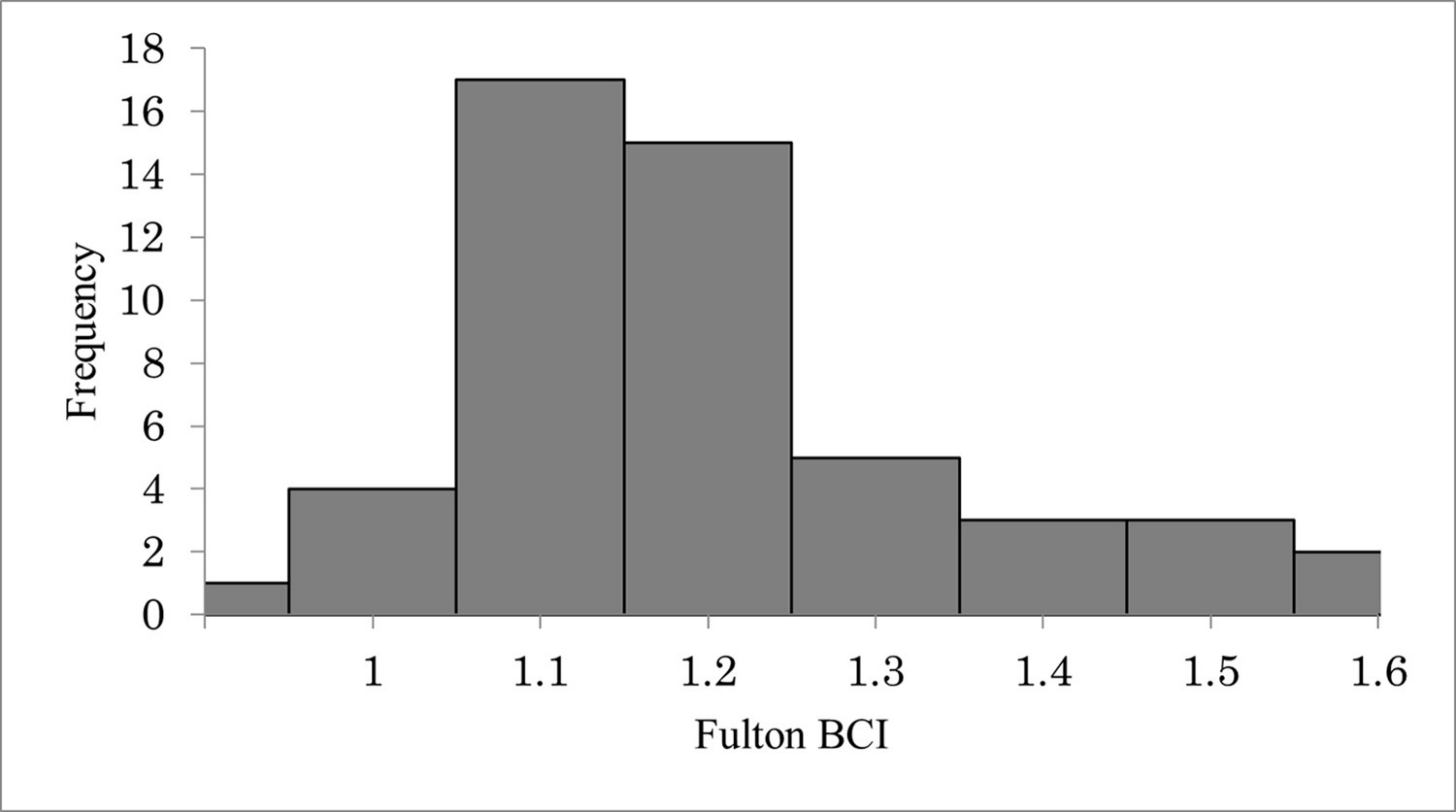
BCI distribution of sea turtles in the study area.

We weighed and calculated the BCI of 49 green turtles captured in 2011, 2014, 2016, and 2019 (Fig 3). The mean BCI for green turtles was 1.35 ± 0.16 (range = 1.02 to 1.83). Fulton’s condition index was not significantly different between maturity stages (chi-square test, χ^2^ = 11.04, df = 6, p > 0.05).

**Fig 4:**
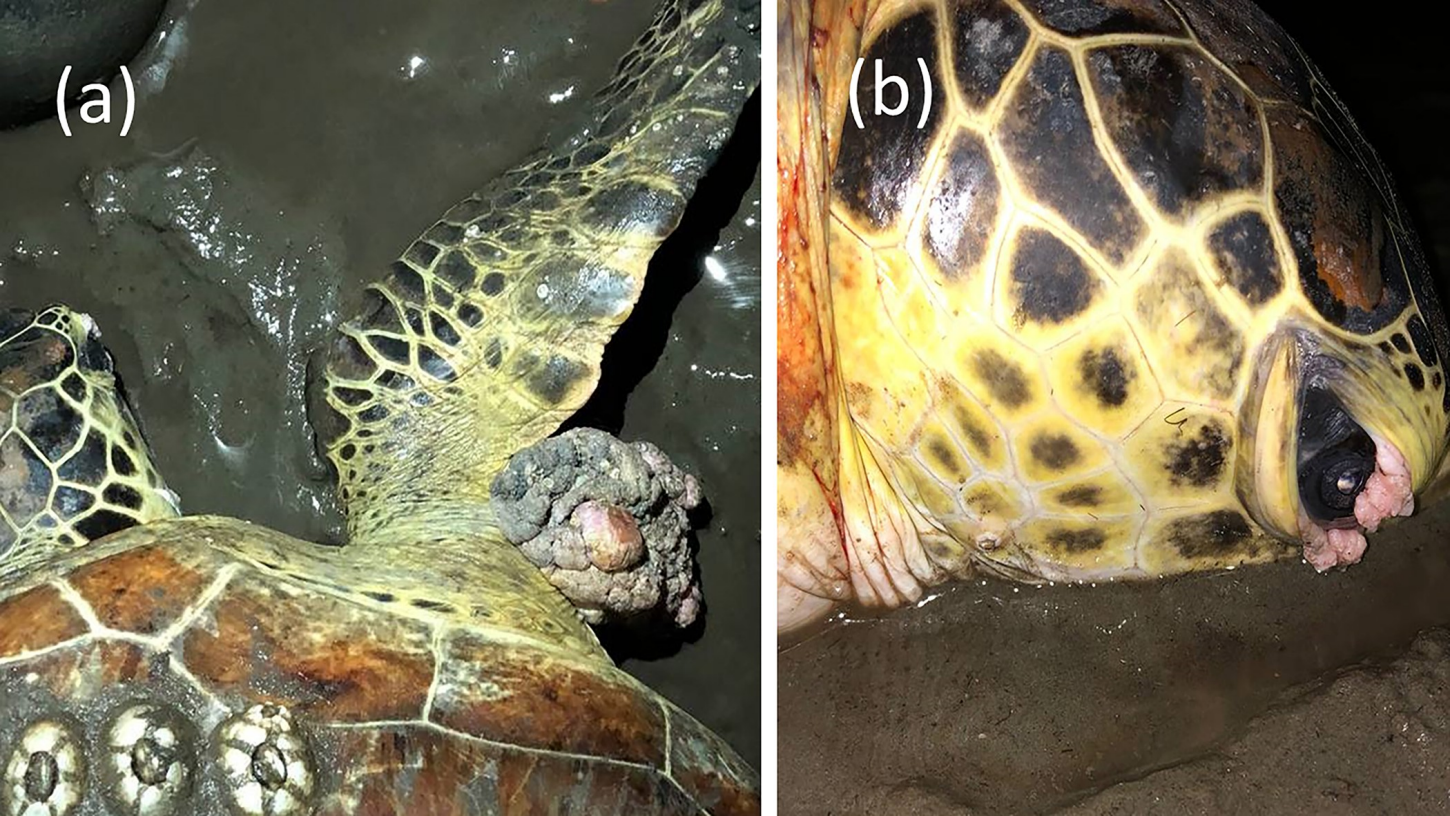
Tumour found on two green turtles (a) on the right flipper and (b) on the right eye.

**Fig 5:**
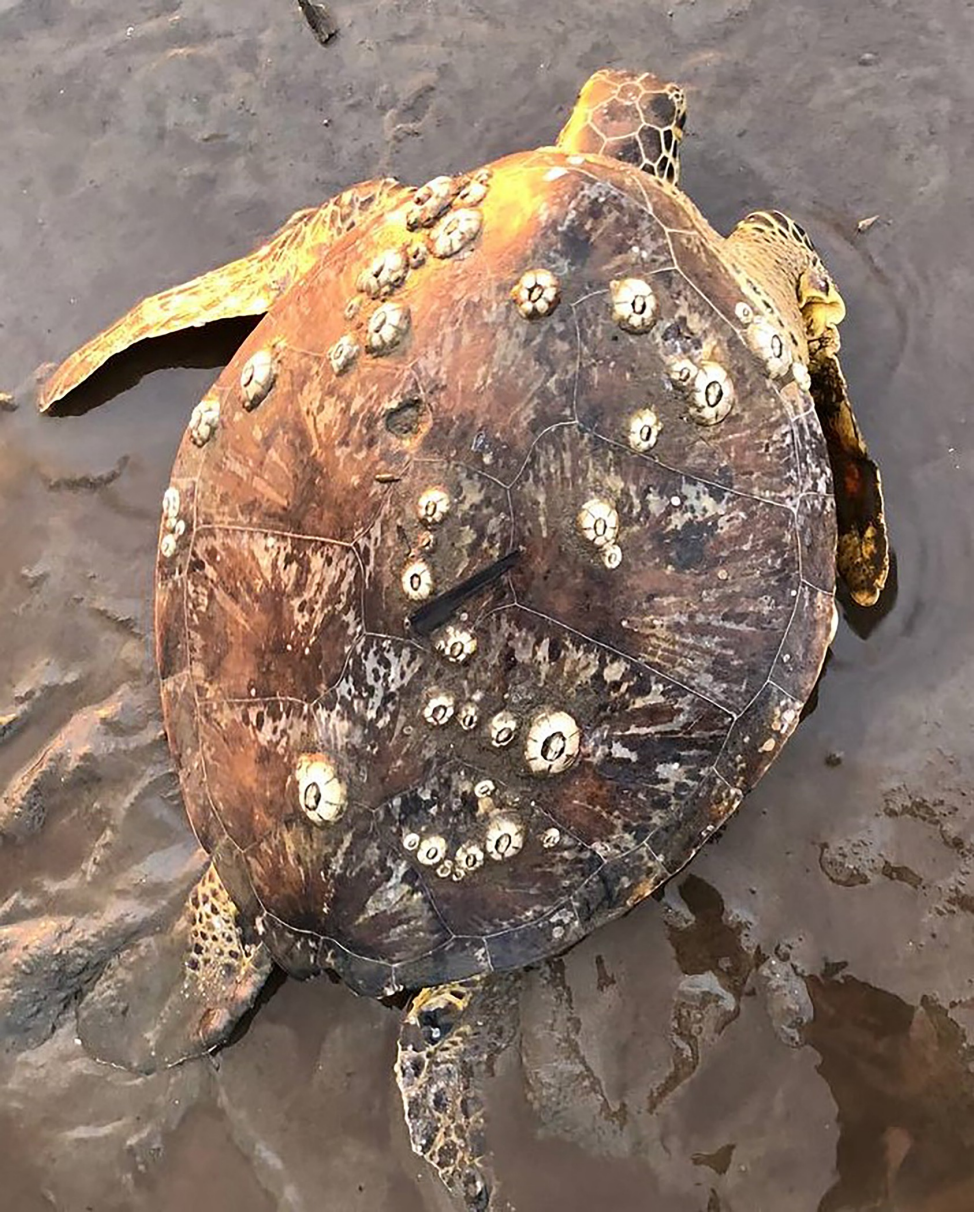
Barnacles (*Chelonibia testudinaria*) attached to the carapace of a green turtle.

Two individuals were diagnosed with mild tumours (score 1) (Fig 4), while all epibionts were identified as *Chelonibia testudinaria* (Fig 5). The number of barnacles per turtle ranged from 8 to 230 individuals (mean = 47 ± 44.4) and was not significantly related to carapace length (F = 0.967, df = 1, 58, p = 0.329). A significant correlation to CCL was observed in MCH (τ = 0.37, p < 0.0001) and MO (τ = -0.36, p = 0.0002) (Table 1). No other significant correlations to CCL or the number of barnacles were observed after sequential Bonferroni corrections. One of the turtles affected by tumour exhibited relatively high WBC (17.5 × 10⁹ / L) and LYM (11.9 × 10⁹ / L). In contrast, the values for another turtle were within the normal range (11.0 × 10⁹ / L and 5.8 × 10⁹ / L, respectively).

**Table 1:** Hematology results for green turtle in Brunei Bay.

Parameter	*N*	Median	10^th^ and 90^th^ percentiles	Correlation to CCL	Correlation to barnacles
**Red blood cell (10^12^/L)**	60	0.420	0.249–0.530	-0.23	-0.09
**Hemoglobin (g/L)**	60	116.0	64.6–136.6	0.00	-0.12
**Hematocrit (L/L)**	57	0.04	0.02–0.06	-0.17	0.11
**Mean cell volume (f/L)**	57	82.0	63.6–135.0	-0.01	0.20
**Mean cell hemoglobin (pg)**	60	271.0	225.8–373.3	0.37*	0.00
**Mean cell hemoglobin concentration (g/dL)**	57	3152.0	2122.4–4546.6	0.13	-0.18
**White blood cell (10⁹/L)**	58	9.90	2.88–15.46	-0.19	-0.05
**Heterophils (10⁹/L)**	60	0.90	0.20–2.13	-0.18	-0.19
**Lymphocytes (10⁹/L)**	60	5.00	0.40–9.01	-0.12	0.02
**Monocytes (10⁹/L)**	60	0.20	0.07–1.10	-0.36*	-0.12
**Eosinophils (10⁹/L)**	60	2.50	0.07–4.40	-0.19	-0.17
**Thrombocytes (10⁹/L)**	60	13.0	2.9–48.1	0.14	0.08
**H: L ratio**	60	0.22	0.09–0.78	-0.01	-0.12

Asterisks indicate significant Kendall rank correlation coefficients.

### Genetic composition

The mtDNA haplotype was successfully determined in 58 green turtles in 2018–2019 in Brunei Bay (Table 2). In total, we identified 11 haplotypes, 10 of which were detected in 2011–2016 [21, 22]. One haplotype was not previously described and was registered in GenBank as CmP20.2 (KF311744).

**Table 2:** Haplotype composition of green turtle aggregations.

Haplotype	GenBank accession no.	New samples	Temporal difference (December–May)			Difference between stages (2011–2019)	
		2018–2019	2011	2013–2014	2018–2019	Immature	Mature
**CmP20.1**	AB819808		1	2		3	
**CmP20.2**	KF311744	1			1		1
**CmP32.1**	KF311749						1
**CmP40.1**	KF311750	1	1	1		2	1
**CmP49.1**	AB819808	14	4	3	8	9	12
**CmP49.3**	KJ502572	1	1	1	1	1	2
**CmP57.1**	KJ502588	7	9	5	5	14	7
**Cmp57.2**	KJ502567	3		1	3	1	4
**CmP75.1**	KJ502574			1		1	
**CmP82.1**	KJ502584	2		1	2		3
**CmP87.1**	KJ502589	17	4	2	14	8	17
**CmP91.1**	KF311750	9	2		4	6	5
**CmP104.1**	KJ502569	2		2	1	2	3
**CmP154.1**	KM923922		1				1
**CmP230.1**	KX057745	1					2
**Total**		58	23	19	39	47	59
**Reference**		This study	21	21	This study	21, 22, This study	21, 22, This study

The haplotype frequency of green turtles was not significantly different among months in 2018 (p > 0.249). Haplotype frequency in 2018–2019 was not significantly different from that in 2011 or 2013–2014 reported previously [21] (p > 0.060; Table 2). There were also no significant differences in haplotype frequency between mature and immature turtles (p = 0.090).

Regardless of whether the prior distribution was uniform or weighted by the number of nests, the MSA showed that the natal origin of immature and mature green turtles was mainly from the South China Sea (95% credible intervals in immature: 22.1–65.1% and 21.6–65.1%, mature: 45.4–80.1% and 40.3–77.1% in uniform and weighted priors, respectively) and in Sulu and Celebes Sea (immature: 25.5–63.5% and 25.7–66.3%, mature: 14.9–45.2% and 16.8–56.6%; Table 3), particularly from Sarawak and Sabah Turtle Islands (S1 Table). The credible intervals overlapped, but the contribution from the South China Sea or Sarawak tended to be higher for mature turtles than for immature turtles.

**Table 3:** Estimated origin (percentage) of (a) immature and (b) mature green turtles in Brunei Bay from five regional groups of rookeries.

(a)										
**Region**	**Uniform prior**					**Weighted prior**				
	**Mean**	**SD**	**2.5%**	**Median**	**97.5%**	**Mean**	**SD**	**2.5%**	**Median**	**97.5%**
**Southern Pacific**	3.4	6.3	0.0	0.2	21.8	1.4	4.4	0.0	0.1	18.5
**Micronesia**	3.6	3.9	0.0	2.4	13.3	0.9	2.5	0.0	0.0	9.2
**South China Sea**	42.7	11.2	22.1	42.3	65.1	41.6	10.9	21.6	40.9	65.1
**Sulu and Celebes Sea**	42.0	9.6	25.5	41.2	63.5	41.7	9.9	25.7	40.6	66.3
**Eastern Indian Ocean**	8.3	8.7	0.0	5.7	28.5	14.4	9.4	0.1	15.1	32.6
(b)										
**Region**	**Uniform prior**					**Weighted prior**				
	**Mean**	**SD**	**2.5%**	**Median**	**97.5%**	**Mean**	**SD**	**2.5%**	**Median**	**97.5%**
**Southern Pacific**	1.1	2.6	0.0	0.1	9.7	0.6	1.9	0.0	0.0	5.5
**Micronesia**	2.5	2.4	0.0	1.9	8.7	0.9	1.8	0.0	0.0	6.2
**South China Sea**	64.8	9.1	45.4	64.4	80.1	58.3	9.4	40.3	58.2	77.1
**Sulu and Celebes Sea**	27.2	7.6	14.9	26.3	45.2	34.7	11.0	16.8	33.7	56.6
**Eastern Indian Ocean**	4.4	5.5	0.0	1.6	18.1	5.5	5.9	0.0	3.0	19.4

## Discussion

Brunei Bay is an important foraging ground for green turtles. Large juvenile and adult green turtles mainly used Brunei Bay, as reported by Joseph et al. [21]. Brunei Bay has extensive seagrass beds [44] that are suitable habitats for green turtles. Here we found that the size of the aggregation has been relatively constant from 2011 to 2019. Recently, decrease in the size of nesting green turtles has been reported from several nesting sites, possibly due to increase in the neophyte nesters [45–47]. However, significant decrease in the CCL of foraging green turtles in the Brunei Bay was not supported. On the other hand, green turtles captured in August were significantly smaller than those in January and April in 2018, indicating the seasonal change in size composition. Further study is needed for several additional years, but the seasonal change may be due to the reproductive migration of adults.

The relatively stable composition of the foraging aggregation in Brunei Bay was supported by genetic analysis and non-significant differences in haplotype composition among seasons and years. As suggested by Joseph et al. [21] and Nishizawa et al. [22], green turtles in Brunei Bay mainly originated from nearby rookeries in Sarawak and Sabah Turtle Islands Parks. On the other hand, despite the overlap of the credible intervals, the contribution from the nearby Sarawak rookery to mature turtles tended to be higher. This suggests that the possible migration of older juveniles to the foraging grounds near their natal beach [48].

The body condition of foraging green turtles in Brunei Bay was relatively good, as Fulton’s BCI was within the range of apparently healthy green turtles (1.25–1.36 for small juveniles, 1.34–1.47 for large juveniles and adults) [17]. Mildly affected tumours were observed from only two individuals; thus, the fibropapilomatosis is less prevailed in Brunei Bay in comparison with Indonesia [5]. Loading of the barnacle *C*. *testudinaria* was higher in Brunei Bay than in subtropical and temperate areas [49, 50]. However, the abundance of barnacles is remarkable and comparable to that in Mabul Island, Sabah, Malaysia [51]. The high abundance of barnacles may not signify unhealthy green turtles, but rather reflects the unique dynamics of the bay, where the muddy water may contain many organic matters that support a high abundance of barnacles.

In general, the blood reference intervals of Brunei Bay green turtles are within those reported for clinically healthy green turtles from San Diego Bay [12], Australia [52], Palmyra Atoll [53], and Galapagos [54]. WBC and LYM of a tumour-affected individual were relatively high, in contrast with Work and Balazs [34] which reported a decrease in these parameters as tumour scores increased. However, such characteristics were not observed in another tumour-affected individual; thus, the effect of tumours on blood parameters was not evident in this study. As Stamper et al. [55] reported, we detected no significant correlations between epibiotic load and blood parameters. The H: L ratio is an indicator of physiological stress in vertebrates including reptiles [36], but was not significantly correlated with the number of *C*. *testudinaria*, indicating that their attachment is not a stress factor for green turtles.

Blood parameters of sea turtles are thought to be related to age and migratory status [16, 55, 56]. Higher MCH in larger green turtles in this study is in accordance with higher erythropoietic parameters in adults than juveniles in loggerhead turtles [16] and other aquatic reptiles [57, 58], indicating adaptation for higher oxygen supply and diving capability in larger individuals. On the other hand, there was a negative correlation between CCL and MO. MO is phagocytic cells associated with defence against infections and bacteria [36] and known to increase with tumour scores [34]. Thus, smaller green turtles might be more exposed to inflammatory factors. However, MO values in the Brunei Bay are relatively low in comparison with previous studies [12, 52–54]. MO can vary geographically, seasonally or with reproductive status (for example, lower MO in nesting leatherback turtles than foraging turtles [56]).

## Conclusion

Under the development of coastal areas and changes in coastal ecosystems, establishing baseline data on the health status and body condition of foraging green turtles is important. Since Brunei Bay remains relatively unpolluted [25, 26] and biodiverse [23, 24], the values of hematology parameters and Fulton’s body condition index in this study will be referred to as baseline data. This study also indicated a seasonal change in size composition possibly related to reproduction. However, fluctuations of hematology parameters depending on seasons and reproductive status have not been fully understood in sea turtles; thus, further studies about these fluctuations will be helpful for monitoring the health status and body condition of foraging sea turtles. Monitoring foraging aggregations regularly in combination with individual identification (e.g., photo-identification [59]) will be important in further studies.

## Supporting information

S1 Table Estimated origin (percentage) of (a) immature and (b) mature green turtles in the Brunei Bay from 32 management units of nesting populations. (DOCX)
