# Supplementary material for: Health status and genetic compositions of green turtles (Chelonia mydas) foraging in Brunei Bay
Source: PLoS One. 2023 Nov 9;18(11):e0293979. doi: 10.1371/journal.pone.0293979 (PMC10635546; doi:10.1371/journal.pone.0293979)
Supplement: S1 Table — (DOCX) [file pone.0293979.s001.docx]

Supplementary material: Estimated origin (percentage) of (a) immature and (b) mature green turtles in the Brunei Bay from 32 management units of nesting populations.

| (a) | Uniform prior | | | | | Weighted prior | | | | |
| --- | --- | --- | --- | --- | --- | --- | --- | --- | --- | --- |
| Source rookery | Mean | SD | 2.5% | Median | 97.5% | Mean | SD | 2.5% | Median | 97.5% |
| nGBR | 0.1 | 0.4 | 0.0 | 0.0 | 0.8 | 0.3 | 0.7 | 0.0 | 0.0 | 2.4 |
| Coral Sea | 0.1 | 0.4 | 0.0 | 0.0 | 0.8 | 0.0 | 0.3 | 0.0 | 0.0 | 0.2 |
| sGBR | 0.1 | 0.4 | 0.0 | 0.0 | 0.7 | 0.1 | 0.4 | 0.0 | 0.0 | 0.8 |
| wNew Caledonia | 0.1 | 0.4 | 0.0 | 0.0 | 0.8 | 0.0 | 0.2 | 0.0 | 0.0 | 0.1 |
| nNew Guinea | 1.0 | 2.7 | 0.0 | 0.0 | 9.5 | 0.6 | 2.2 | 0.0 | 0.0 | 7.9 |
| Vanuatu | 3.0 | 6.3 | 0.0 | 0.0 | 21.4 | 1.0 | 4.3 | 0.0 | 0.0 | 18.1 |
| Micronesia | 0.3 | 1.2 | 0.0 | 0.0 | 3.9 | 0.1 | 0.8 | 0.0 | 0.0 | 0.2 |
| MarshallIs | 0.5 | 1.7 | 0.0 | 0.0 | 6.3 | 0.1 | 0.9 | 0.0 | 0.0 | 0.1 |
| Palau | 0.7 | 2.0 | 0.0 | 0.0 | 7.4 | 0.1 | 0.9 | 0.0 | 0.0 | 0.0 |
| CNMI / Guam | 1.1 | 2.6 | 0.0 | 0.0 | 9.3 | 0.0 | 0.4 | 0.0 | 0.0 | 0.0 |
| Samoa | 0.1 | 0.4 | 0.0 | 0.0 | 0.8 | 0.0 | 0.0 | 0.0 | 0.0 | 0.0 |
| French Polynesia | 0.1 | 0.4 | 0.0 | 0.0 | 0.8 | 0.0 | 0.1 | 0.0 | 0.0 | 0.0 |
| Penang & Perak | 0.4 | 1.7 | 0.0 | 0.0 | 5.0 | 0.0 | 0.3 | 0.0 | 0.0 | 0.0 |
| Vietnam | 0.3 | 1.3 | 0.0 | 0.0 | 3.3 | 0.0 | 0.4 | 0.0 | 0.0 | 0.0 |
| Perhentian | 0.3 | 1.5 | 0.0 | 0.0 | 3.9 | 0.0 | 0.2 | 0.0 | 0.0 | 0.0 |
| Redang | 0.5 | 2.4 | 0.0 | 0.0 | 6.1 | 0.1 | 1.0 | 0.0 | 0.0 | 0.0 |
| Terengganu | 11.7 | 8.0 | 0.0 | 10.6 | 29.9 | 18.1 | 14.9 | 0.0 | 15.6 | 57.7 |
| Pahang | 0.2 | 1.1 | 0.0 | 0.0 | 2.2 | 0.0 | 0.2 | 0.0 | 0.0 | 0.0 |
| Mersing | 6.3 | 9.0 | 0.0 | 0.0 | 28.5 | 0.9 | 5.4 | 0.0 | 0.0 | 16.4 |
| Sarawak | 23.0 | 7.8 | 9.4 | 22.5 | 39.4 | 22.5 | 10.8 | 0.0 | 22.9 | 42.8 |
| STIP | 34.8 | 10.1 | 9.6 | 35.5 | 52.0 | 37.4 | 8.6 | 20.5 | 37.6 | 53.3 |
| Sipadan | 6.3 | 12.8 | 0.0 | 0.0 | 46.5 | 1.6 | 8.3 | 0.0 | 0.0 | 28.5 |
| Breau Is | 1.0 | 3.4 | 0.0 | 0.0 | 11.8 | 2.7 | 7.0 | 0.0 | 0.0 | 26.6 |
| Aru | 5.6 | 7.3 | 0.0 | 0.7 | 22.6 | 12.0 | 8.7 | 0.0 | 12.8 | 28.5 |
| GOC | 0.1 | 0.6 | 0.0 | 0.0 | 1.2 | 0.1 | 0.7 | 0.0 | 0.0 | 1.4 |
| Ashmore Reef | 1.2 | 3.5 | 0.0 | 0.0 | 13.0 | 0.6 | 2.8 | 0.0 | 0.0 | 10.2 |
| Scott | 0.2 | 1.2 | 0.0 | 0.0 | 2.6 | 0.0 | 0.4 | 0.0 | 0.0 | 0.0 |
| West Java | 0.6 | 2.2 | 0.0 | 0.0 | 7.8 | 0.1 | 0.8 | 0.0 | 0.0 | 0.0 |
| NW Shelf | 0.1 | 0.4 | 0.0 | 0.0 | 0.9 | 1.6 | 2.0 | 0.0 | 0.9 | 7.1 |
| Cobourg Peninsula | 0.1 | 0.5 | 0.0 | 0.0 | 1.0 | 0.0 | 0.2 | 0.0 | 0.0 | 0.0 |
| Cocos Keeling | 0.4 | 1.8 | 0.0 | 0.0 | 5.1 | 0.0 | 0.6 | 0.0 | 0.0 | 0.0 |
| Xisha Islands | 0.1 | 0.5 | 0.0 | 0.0 | 1.1 | 0.0 | 0.1 | 0.0 | 0.0 | 0.0 |

| (b) | Uniform prior | | | | | Weighted prior | | | | |
| --- | --- | --- | --- | --- | --- | --- | --- | --- | --- | --- |
| Source rookery | Mean | SD | 2.5% | Median | 97.5% | Mean | SD | 2.5% | Median | 97.5% |
| nGBR | 0.1 | 0.3 | 0.0 | 0.0 | 0.6 | 0.2 | 0.7 | 0.0 | 0.0 | 2.1 |
| Coral Sea | 0.1 | 0.3 | 0.0 | 0.0 | 0.6 | 0.0 | 0.2 | 0.0 | 0.0 | 0.2 |
| sGBR | 0.1 | 0.3 | 0.0 | 0.0 | 0.6 | 0.1 | 0.3 | 0.0 | 0.0 | 0.7 |
| wNew Caledonia | 0.1 | 0.3 | 0.0 | 0.0 | 0.6 | 0.0 | 0.2 | 0.0 | 0.0 | 0.1 |
| nNew Guinea | 0.1 | 0.4 | 0.0 | 0.0 | 0.7 | 0.0 | 0.2 | 0.0 | 0.0 | 0.0 |
| Vanuatu | 0.7 | 2.4 | 0.0 | 0.0 | 9.3 | 0.3 | 1.7 | 0.0 | 0.0 | 3.0 |
| Micronesia | 1.0 | 1.8 | 0.0 | 0.0 | 6.2 | 0.7 | 1.6 | 0.0 | 0.0 | 5.7 |
| MarshallIs | 0.4 | 1.1 | 0.0 | 0.0 | 3.6 | 0.1 | 0.4 | 0.0 | 0.0 | 0.5 |
| Palau | 1.0 | 1.6 | 0.0 | 0.1 | 5.5 | 0.1 | 0.7 | 0.0 | 0.0 | 2.1 |
| CNMI / Guam | 0.1 | 0.3 | 0.0 | 0.0 | 0.7 | 0.0 | 0.0 | 0.0 | 0.0 | 0.0 |
| Samoa | 0.1 | 0.4 | 0.0 | 0.0 | 0.7 | 0.0 | 0.0 | 0.0 | 0.0 | 0.0 |
| French Polynesia | 0.1 | 0.4 | 0.0 | 0.0 | 0.8 | 0.0 | 0.1 | 0.0 | 0.0 | 0.0 |
| Penang & Perak | 0.5 | 2.1 | 0.0 | 0.0 | 7.2 | 0.0 | 0.2 | 0.0 | 0.0 | 0.0 |
| Vietnam | 0.3 | 1.3 | 0.0 | 0.0 | 3.4 | 0.0 | 0.6 | 0.0 | 0.0 | 0.0 |
| Perhentian | 0.6 | 2.3 | 0.0 | 0.0 | 8.0 | 0.0 | 0.5 | 0.0 | 0.0 | 0.0 |
| Redang | 2.8 | 7.1 | 0.0 | 0.0 | 26.5 | 1.3 | 6.0 | 0.0 | 0.0 | 23.5 |
| Terengganu | 3.5 | 6.8 | 0.0 | 0.0 | 23.5 | 1.0 | 4.8 | 0.0 | 0.0 | 18.4 |
| Pahang | 0.2 | 1.3 | 0.0 | 0.0 | 2.8 | 0.0 | 0.3 | 0.0 | 0.0 | 0.0 |
| Mersing | 6.3 | 7.1 | 0.0 | 4.4 | 22.7 | 1.3 | 4.6 | 0.0 | 0.0 | 18.1 |
| Sarawak | 49.5 | 9.9 | 29.4 | 49.9 | 67.8 | 54.5 | 9.4 | 34.0 | 55.2 | 71.1 |
| STIP | 22.8 | 7.4 | 1.2 | 23.0 | 36.5 | 22.2 | 6.7 | 10.3 | 21.9 | 36.1 |
| Sipadan | 2.7 | 8.1 | 0.0 | 0.0 | 32.5 | 0.2 | 2.2 | 0.0 | 0.0 | 0.0 |
| Breau Is | 1.7 | 4.8 | 0.0 | 0.0 | 18.0 | 12.3 | 12.4 | 0.0 | 10.4 | 36.9 |
| Aru | 3.0 | 4.6 | 0.0 | 0.0 | 14.8 | 4.0 | 5.5 | 0.0 | 0.0 | 16.9 |
| GOC | 0.1 | 0.5 | 0.0 | 0.0 | 1.0 | 0.1 | 0.7 | 0.0 | 0.0 | 1.4 |
| Ashmore Reef | 0.1 | 0.6 | 0.0 | 0.0 | 1.3 | 0.0 | 0.2 | 0.0 | 0.0 | 0.0 |
| Scott | 0.2 | 1.0 | 0.0 | 0.0 | 2.1 | 0.0 | 0.3 | 0.0 | 0.0 | 0.0 |
| West Java | 0.3 | 1.4 | 0.0 | 0.0 | 3.9 | 0.0 | 0.3 | 0.0 | 0.0 | 0.0 |
| NW Shelf | 0.1 | 0.4 | 0.0 | 0.0 | 0.7 | 1.4 | 1.7 | 0.0 | 0.8 | 6.2 |
| Cobourg Peninsula | 0.1 | 0.5 | 0.0 | 0.0 | 0.9 | 0.0 | 0.1 | 0.0 | 0.0 | 0.0 |
| Cocos Keeling | 0.5 | 2.0 | 0.0 | 0.0 | 6.5 | 0.1 | 0.8 | 0.0 | 0.0 | 0.0 |
| Xisha Islands | 1.1 | 1.7 | 0.0 | 0.4 | 5.9 | 0.1 | 0.6 | 0.0 | 0.0 | 1.1 |
